# From pandas to pans: a nosographic entity in evolution throughout a descriptive analysis of a cohort of 103 italian children and adolescents

**DOI:** 10.1186/1546-0096-12-S1-P300

**Published:** 2014-09-17

**Authors:** Fernanda Falcini, Gemma Lepri, Federico Bertini, Marco Matucci Cerinic, Donato Rigante

**Affiliations:** 1 Department of BioMedicine, Section of Rheumatology, Transition Clinic, University of Florence, Florence, Italy; 2 Istitute of Pediatrics, Univeristy Cattolica del Sacro Cuore, Rome, Italy

## Introduction

The acronym PANDAS reveals a pediatric autoimmune neuropsychiatric disorder associated with *Streptococcus pyogenes* infection. This disorder is defined by: **(a)** the presence of obsessive-compulsive disorder (OCD) or tics; **(b)** abrupt onset and remitting/relapsing course; **(c)** pre-puberal onset of all symptoms; **(d)** temporal association with a group A β-hemolytic *S. pyogenes* infection; **(e)** association with neurologic disorders (hyperactivity, choreiform movements, etc). In addition to PANDAS, another pediatric neropsychiatric syndrome with an abrupt onset is now recognized. This disorder is called PANS and is characterized by different neuropsychiatric manifestations, which seem to be temporally associated with infectious agents different from *S. pyogenes* or with other environmental triggers.

## Objectives

To describe clinical features of an Italian cohort of children and adolescents with PANDAS and PANS and to highlight differences between the two groups.

## Methods

From May 2009 to May 2014, 103 Italian patients (78 M, 25 F), mean age 117,7±41,5 months, presenting OCD and/or tics (vocal and/or motor tics) starting before puberty, were enrolled in this observational study. Demographic and familiar data were collected for all patients, as well as routine and specific laboratory data, including thyroid function, TAOSL (anti-streptolysin O titer), serology for Cytomegalovirus virus, Epstein-Barr virus, *Mycoplasma pneumoniae*, *Borrelia burdorferi*, and autoimmunity tests (ANA, anti-dsDNA, anti-ENA, anti-cardiolipin, anti-tissue transglutaminase antibodies and LAC). In addition, a sub cohort of patients was investigated with brain MRI, EEG, and echocardiography. Patients were also evaluated by a neuropsychiatrist.

## Results

Fifty eight/103 patients (46.6%) had other relatives with established OCD/tics or other neurologic disease**.** All subjects presented an abrupt, acute onset; mean age of symptom’ onset was 77,2±27,6 months. In our cohort, the mean age at diagnosis was 102,8±32,7 months. In 62 (60,2%) patients, pharyngitis, otitis and/or upper airway infections were previously reported (1/62 presented impetigo). In 96 (93%), the TAOSL was increased (500-800 IU/ml), while the anti-DNase titer was between 650 and 1200 IU. It was not possible to demonstrate a *S. pyogenes* infection in 7/103 patients (6,5%). Out of these whom, 3 presented a recent Ebstein Barr virus infection, 3 had signs of a recent *M. pneumonia* infection and in 1 patient a recent *B. burdorferi* infection was demonstrated. As specified in “patients and method”, 77 patients were evaluated by MRI, EEG, and echocardiography, which were normal in all subjects. All patients were treated with amoxicilline and benzathine benzylpenicillin. Sixty nine patients (66,9%) showed a complete or partial remission of the initial symptoms.

## Conclusion

Our data confirm that patients with PANDAS present an acute abrupt clinical onset at mean age of 6±2 years. This neuropsychiatric disorder is mainly observed in males, commonly having other relatives suffering from neuropsychiatric disorders (about the 50% of them). Seven patients did not present evidence of *S. pyogenes* infection, and these patients may be collocated in the PANS group. Antibiotic therapy seemed to be efficacious also in the latter patients, with a complete or partial remission of symptoms. Our data highlight the close clinical similarity between PANDAS and PANS. In clinical practice, these two disorders may be distinguishable only using laboratory test with the aim of identifying their etiologic agents.

## Disclosure of interest

None declared.

